# Rapid detection of vaccinia virus using biofunctionalized fiber-optic ball-tip biosensors

**DOI:** 10.1038/s41598-023-44926-6

**Published:** 2023-10-14

**Authors:** Aida Rakhimbekova, Baizak Kudaibergenov, Kuanysh Seitkamal, Aurora Bellone, Ayazhan Dauletova, Marzhan Sypabekova, Massimo Olivero, Guido Perrone, Antonia Radaelli, Carlo Zanotto, Carlo De Giuli Morghen, Luca Vangelista, Daniele Tosi

**Affiliations:** 1 Department of Electrical and Computer Engineering, School of Engineering and Digital Sciences, Nazarbayev University, 010000 Astana, Kazakhstan; 2 Department of Biomedical Sciences, School of Medicine, Nazarbayev University, 010000 Astana, Kazakhstan; 3 Department of Electronics and Telecommunications, Politecnico Di Torino, Turin, Italy; 4 Department of Electrical & Computer Engineering, Baylor University, Waco, TX USA; 5 Department of Medical Biotechnologies and Translational Medicine, Laboratory of Molecular Virology and Recombinant Vaccine Development, University of Milan, Via Vanvitelli 32, Milan, Italy; 6 Catholic University “Our Lady of Good Counsel”, Rr. Dritan Hoxha, Tirana, Albania; 7 Department of Molecular Medicine, University of Pavia, 27100 Pavia, Italy; 8 Laboratory of Biosensors and Bioinstruments, National Laboratory Astana, 010000 Astana, Kazakhstan

**Keywords:** Biomedical engineering, Applied optics, Infectious-disease diagnostics

## Abstract

In this work, we present the development and biofunctionalization of a fiber-optic ball-resonator biosensor for the real-time detection of vaccinia poxvirus. We fabricated several ball-tip resonators, functionalized through a silanization process to immobilize two bioreceptors: the monoclonal anti-L1R antibody targeting the L1R protein, and the polyclonal rabbit serum antibodies targeting the whole vaccinia virus (VV) pathogen. Experimental measurements were carried out to detect VV in concentrations from 10^3^ to 10^8^ plaque-forming units (PFU), with a limit of detection of around 1.7–4.3 × 10^3^ PFU and a log-quadratic pattern, with a response up to 5 × 10^−4^ RIU (refractive index units). The specificity was assessed against herpes simplex virus, used as a non-specific control, with the best results obtained with anti-L1R monoclonal antibodies, and through the detection of vaccinia virus/herpes simplex-1 combination. The obtained results provide a real-time viral recognition with a label-free sensing platform, having rapid response and ease of manufacturing, and paving the road to the seamless detection of poxviruses affecting different human and animal species using optical fibers.

## Introduction

The recent COVID-19 pandemic has brought a substantial attention on the development of biosensing technologies that can rapidly and reliably detect the presence of viral pathogens in liquid media^1^. The ultimate goal is to provide effective platforms that can detect viruses in real time, with high specificity, and with the possibility to enable large-scale measurements over array of sensors^2^. In parallel to traditional immunoassays that are commonly used in medical laboratories^3^, the possibility to use biosensors based on electrochemical^4^, optical^5^, plasmonic^6^, and optical fiber^7^ technologies provide an additional improvement towards speed of detection, affordability, and versatility, coping with the possibility of diagnostic in multiple environments. Among others, Barceló^8^ and Schmidt^9^ proposed real-time sensing methods operating in wastewater environment as a tool for surveillance for pandemic outbreaks of pathogens.

Virus agents, and in particular orthopoxviruses (OPXVs), share the common attributes of high morbidity, potential large diffusion, and relatively-easy development as bioweapons for terrorist attacks. Viruses are the most effective agents and were often employed as bioweapons in the last centuries^10–12^. They are more lethal than chemical weapons, more difficult to detect than chemical or nuclear weapons, and their production is less expensive, as basic technologies are available in any biological laboratory. In particular, Variola virus (VARV), the etiological agent of smallpox (at present stored in laboratories of USA and Russia), is a Category A biological weapon, that uses humans as exclusive host and must be considered a high-risk agent for bioterrorism. Although officially declared eradicated in 1980^13^, the human population would be largely unprotected in the case of a smallpox outbreak and a recent technology developed to reconstitute poxviruses raises the threat to a higher level^14^. Monkeypox virus (Mpox) has also recently been reported as a possible pandemic threat due to several outbreaks in non endemic countries^15^. Not least, given the 600-fold increase in the number of human Mpox infections in the Democratic Republic of the Congo (DRC) in the second half of 2013 and the inter-human transmission in 50% of the cases, Mpox is considered a dangerous infectious virus which demands for a rapid and reliable detection system^16^.

Vaccinia virus (VV) protein repertoire shares a very high degree of homology with both variola virus, the causative agent of smallpox, and Mpox^17,18^. Smallpox is a category A agent (stored in laboratories in USA and Russia) and a dreadful pandemic virus completely eradicated in 1980^13^. At present, the human population would be largely unprotected in the case of a smallpox outbreak and a recent technology developed to reconstitute poxviruses raises the threat to a higher level^14^. Mpox has recently been reported as a possible pandemic threat due to several outbreaks in non endemic countries^15^. Therefore, in view of a modern biosecurity development, the possibility to detect complete vaccinia virus particles represents an effective way to set a surveillance against smallpox and Mpox release and spread in deliberate, accidental or natural ways.

Optical fiber biosensors (OFBs) are a modern and effective tool for the detection of biomolecules^19^. OFBs have been employed in the detection of biomarkers^20,21^, cells^22^, and glucose^23^ among others. OFBs are immune to electromagnetic interference, have a microscropic footprint, are inherently biocompatible and fire-safe, and can operate in liquid media. Consistently with their capabilities, the use of OFBs has been recently demonstrated in the detection of COVID-19 related pathogens. In an OFB label-free structure, a sensing device is integrated into the optical fiber and used to detect changes in the refractive index of the surrounding environment, while the same fiber is used to transfer the information to the detector. A biofunctionalization process, usually performed through deposition of a metallic thin film or a silanization process, is then used to immobilize a bioreceptor on the surface of the fiber, forming a selective sensor^24^. Cennamo et al.^7^ reported a fiber-optic sensor for SARS-CoV-2 biorecognition based on a D-shaped plastic optical fiber using a polymeric receptor for the virions. Hadi and Khurshid^25^ reported sensors that exploit a similar detection protocol, using a U-shaped polymer optical fiber, functionalized through monoclonal antibodies immobilized by means of gold nanoparticles on the U-section of the fiber. Yang et al.^26^ proposed a fluorescence-labeled fiber-optic sensor for the detection of SARS-CoV-2 and Influenza A pathogens.

Hence, the vast majority of OFB-based technologies reported for the detection of a virus rely on plasmonic effects over largely multi-mode fibers (MMFs) and usually work in the UV–visible wavelength range. This approach provides high sensitivity, but is quite incompatible with the large-scale, long-term, and seamlessly deployable sensing networks foreseen as a watchdog for pandemic outbreaks, as proposed by Barceló^8^ and Daughton^27^. The main reasons are that MMFs do not maintain a very stable propagation due to random power transfer among modes. In addition, plasmonic biosensors usually operate in transmission, limiting the practicality of the implementation and reducing the possibility to multiplex sensors^28^.

A more efficient approach may be brought by single-mode fiber (SMF) devices, exploiting the availability of telecom fibers and components. SMFs have a very robust power handling, as only the fundamental mode is responsible for light propagation. A variety of biosensors working at telecom wavelengths have been proposed, such as long-period^29^ and tilted^30^ gratings, as well as micro-fiber interferometers^20^, all of them exhibiting high performances. However, the fabrication of such sensors is often cumbersome and not suitable for a mass-production process^31^.

Fiber-tip ball resonators (BRs) are an excellent candidate for biosensing, as they take advantage of the favorable properties of single mode fibers (SMF), and they are made by a rapid fabrication process that requires around one minute with a CO_2_ laser splicer. BRs have been first proposed by Shaimerdenova et al.^32^. A fiber-tip BR acts as a very weak reflector, with a spectral fingerprint that exhibits both an intensity change and a slight wavelength shift when the surrounding refractive index varies. Further studies showed that the polarization-sensitive BR interrogation leads to a higher sensitivity^33^, and proved the detection of cancer biomarkers at attomolar levels using biofunctionalized versions of BRs^21^.

In this work, we demonstrate for the first time the detection of a putative pandemic orthopoxvirus using BR sensors biofunctionalized with antibodies, providing a rapid and specific device. The proof of concept is implemented using VV as a surrogate pathogenic target, and two different bioreceptors, either addressed to the whole virus particle or to its L1R surface protein antigen. The obtained results, in terms of sensitivity, detection limits, and specificity pave the way to the real-time detection of pandemic viruses through fiber-optic sensors having a large versatility and potential for larger sensing networks.

## Materials and methods

### Materials

Monoclonal anti-VV L1R antibodies (NR-417, BEI Resources, NIAID, NIH) and polyclonal rabbit serum antibodies produced at the State University of Milano were employed to detect vaccinia virus.

SMF (Corning SMF-28e; ITU-T G.652.D) optical fiber was used to fabricate the sensors. A venous catheter (size G16) was employed as packaging for the BR sensor.

Sucrose, 70% sulfuric acid: 30% hydrogen peroxide, 3-aminopropyl trimethoxysilane (APTMS), methanol, glutaraldehyde, Bovine serum albumin (BSA), Phosphate buffered saline (PBS), 1 ml (Bioject® Budget 100 IU insulin) were used as reagents during the experiments. All reagents were purchased from Sigma-Aldrich (Darmstadt, Germany).

The mouse monoclonal antibody against the recombinant L1R protein was obtained through the Biodefense and Emerging Infections Research Resources Repository (BEI Resources (NIAID, NIH).

### Synthesis of the bioreceptors

The polyclonal serum antibodies were carried out as follows. Specific Pathogen Free (SPF) 6-week-old rabbits (Charles River Laboratories, Como, Italy) were immunized with chemically inactivated VV (Sclavo strain, used for human vaccination). Virus inactivation was performed by UV and psoralen. In brief, the virus (8 × 10^8^ PFU/ml; PFU = plaque forming units) was mixed with a final psoralen concentration of 10 μg/ml. The mixture was incubated on a 6-well dish for 10 min at room temperature (1 ml/well) under UV lamps (366 nm) at 2 cm distance for 12 min (3 min each time with 1 min interval). Inactivation was tested by viral titration to verify the absence of infectious virus.

Intradermal injections were performed on the back in 4–6 points. The animals were boosted every two weeks during the first month with 2 × 10^8^ PFU/rabbit, and once a month for the following periods, twice with 4 × 10^8^ PFU/rabbit and twice with 1 × 10^8^ PFU/rabbit. Bleedings were performed just before each boost. The viral inoculum was diluted in 500 μl phosphate-buffered saline without Ca^2+^ and Mg^2+^ (PBS^−^).

Boosts were repeated every 2 weeks and animals were bled each time from the median artery of the ear. Blood was then centrifuged and serum collected, aliquoted and frozen. The presence of specific antibodies was verified by ELISA test. Sera were then pooled and purified using HiTrap Protein A and protein G HP columns, both under reducing and non-reducing conditions, and desalting in PBS^-^, following the manufacturer instructions (Sigma Merck Life Science, St Louis, MO).

The plasma fraction was aliquoted and frozen at − 80 °C. The animals were monitored during the whole treatment period for signs of disease, and provided with food and water ad libitum until euthanasia. Every effort was made to minimize their suffering. All of the rabbits were maintained according to the Italian National Guidelines of the Italian Ministry of Health (authorized protocol n. 225/2020) that are based on the European Union Directive 2010/63/EU for animal in vivo experiments. Approval for this study was granted by the Ethical Committee of the University of Milan (Authorization 325/2020-PR, 14 April 2020; Protocol 17,791.16.EXT.0, 19 April 2021).

In accordance with the European Union and with the ARRIVE guidelines, rabbits were euthanized by IV administration of a barbiturate (thiopental), under the supervision of a veterinarian of the animal facility.

### Fabrication and calibration of ball resonators

Ball resonators were fabricated from a SMF (SMF-28e, Corning) using a CO_2_ laser splicer (Fujikura LazerMaster LZM-100). The design of BR sensors was carried out by the Fiber Processing Software FPS1.6 (AFL Technologies), which allowed developing a custom recipe for controlling the splicer starting from the geometrical parameters of the BR sensors. This recipe, similar to that presented by Shaimerdenova et al.^32^, consists of a two-step process: (1) two SMF, that have previously been uncoated and cleaved with a diamond blade, are spliced under the laser beam and (2) the laser keeps heating the joint while the fibers are rotated and pushed forward until they break into a ball-shape thanks to surface tension. For these experiments, the fibers were set to feed forward at speed 0.025–0.1 mm/s, while the CO_2_ laser output was set to 380 bits (correspondent to about 5W). For this investigation, we fabricated 7 sensors, having a diameters between 518 and 569 μm. The main data are reported in the Supplementary Materials.

The calibration of BR sensors was carried out by exposing each sensor to different values of refractive index (RI), obtained by different sucrose mixtures. Each calibration was performed in 5 different RI mixtures with RI ranging from 1.34974 to 1.35845 (in steps of 0.00871 refractive index units, RIU). After detecting the most significant spectral features in polarization spectra^33^, we performed a linear regression of the intensity as a function of the refractive index (RI) and estimated the sensitivity. Only sensors with a coefficient of determination R^2^ > 0.95 in RI calibration were then used for the experimental measurements.

### Functionalization of ball resonators

**Figure 1:**
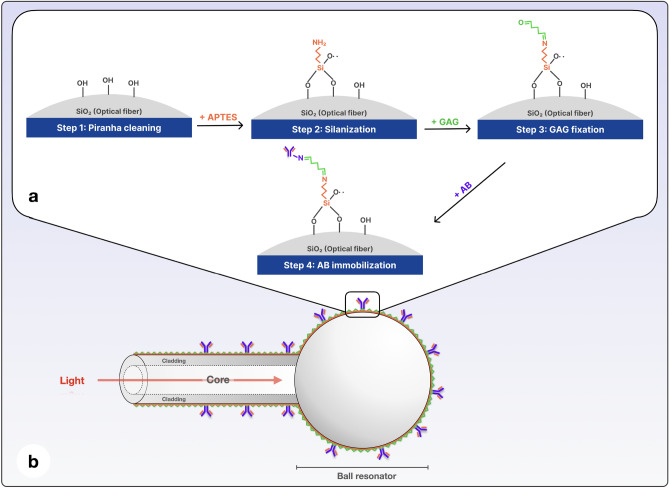
Biofunctionalization process: (**a**) Surface biofunctionalization steps on the fiber surface; (**b**) Schematic of a biofunctionalized fiber optic BR sensor.

The process of biofunctionalization is illustrated in Fig. 1 and hereby detailed. The BR surface was cleaned by placing it in piranha solution (a mixture of sulfuric acid and hydrogen peroxide 4:1) for 15 min at room temperature, which removed organic contaminants and activated the surface of the sensor^34^. Then the surface was washed with deionized water and dried in air. To immobilize the bioreceptor immunoglobulins, the cleaned surface of the BR was treated with a solution of 5% 3-aminopropyl trimethoxysilane (APTMS) in methanol for 90 min at room temperature, and the silanized region was then rinsed with methanol and water. Then, the fibers were inserted in an oven at 80–90 °C for 30 min to eliminate solvents and promote silane cross-linking. The silanized BR sensor was then treated in glutaraldehyde solution (2.5% in PBS) and rinsed with PBS. The fiber was incubated overnight either in 12 µg/ml of monoclonal anti-VV L1R antibodies or in the rabbit serum antibody solution in PBS^21^. On the final stage the surface of BR was rinsed with PBS and used for specific antigen detection.

### Interrogation setup

**Figure 2:**
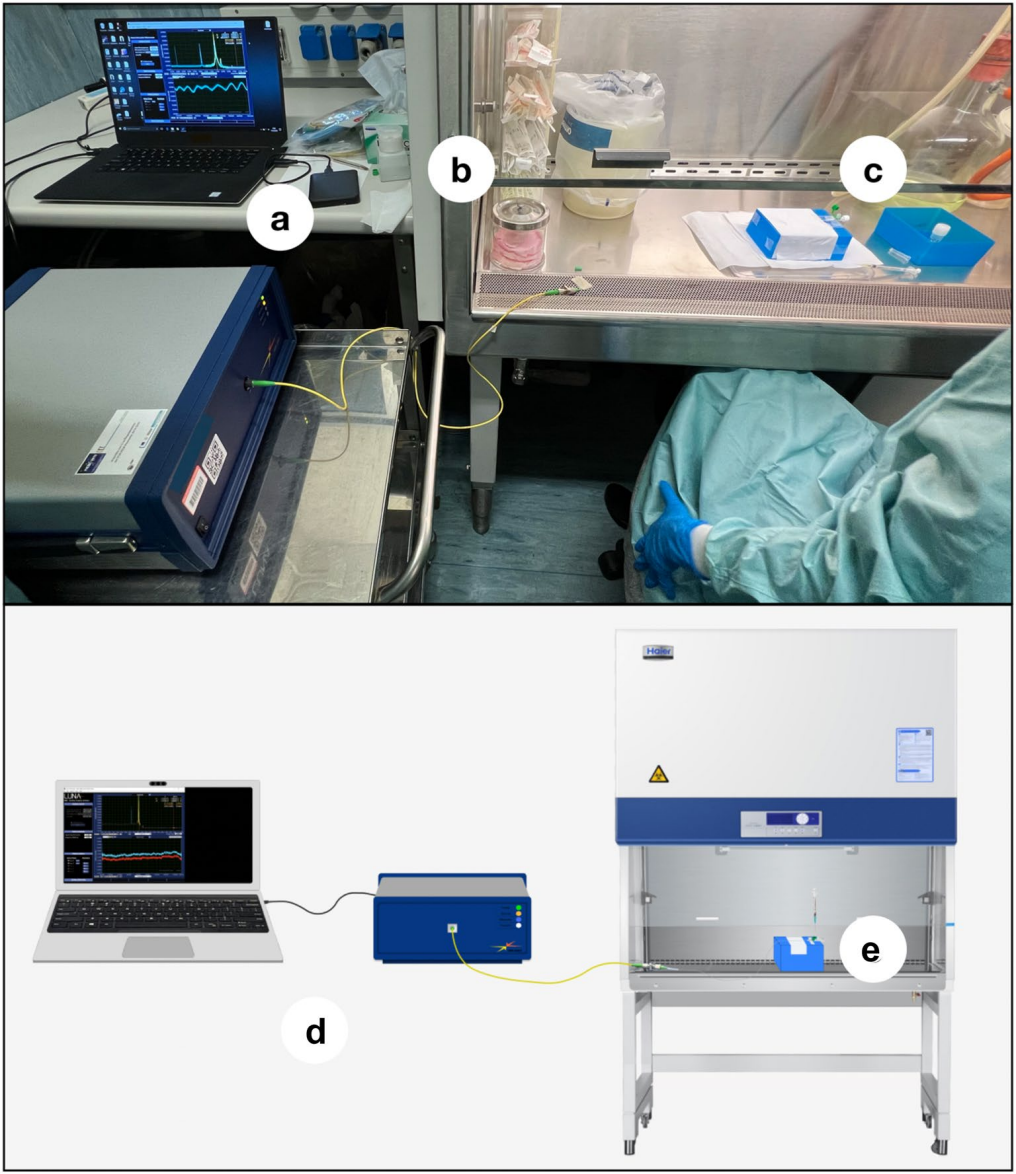
Set up of the experiment: (**a**) Computer used for data acquisition; (**b**) OBR; (**c**) Biological safety cabinet; (**d**) Experimental installation; (**e**) Intravenous catheter fixed on a scaffold box with a biosensor inside and insulin syringe for injection of prepared viral concentrations.

The interrogation of the BRs was was carried out with the polarization-sensitive process described by Shaimerdenova et al., 2021^33^, which tracks the amplitude of the reflection spectrum for either parallel or perpendicular polarization (referred to the laser source of the analyzer). The setup uses a Luna OBR4600 (Luna Inc.) Optical Backscatter Reflectometer (OBR), which uses on a swept-laser interferometer and a polarization beam splitter to extract S-polarization spectra. This method allows a higher sensitivity with respect to the detection of the whole spectrum. For each BR sensor, the most significant spectral feature was identified within the S (perpendicular) polarization. Then the intensity for each peak was tracked using a second-order polynomial fit. This approach was used both for the RI calibration, and for the biological measurements. The instrument was set to acquire spectra in the 1527.215–1612.723 nm range, with a 0.103 GHz resolution bandwidth. Spectra were post-processed using a low-pass finite-impulse response filter (Butterworth, 5th order, cut-off 0.01) to reduce the noise. Figure 2 summarizes the setup through a schematic and some pictures of the devices and tools used in these tests.

### Measurement of virus at various concentrations

Vaccinia Virus (Sclavo), the strain used for human vaccination, was grown in subconfluent Vero cells, then amplified, purified, and titred. Briefly, cells were infected with 0.005 PFU/cell for 1 h at 37 °C and harvested after 5 days. The virus was collected from the supernatant after disrupting the cells several times by sonication, followed by centrifugation at 4 °C for 5 min at 1200 × g. The collected supernatant was then purified on 30–45% w/w discontinuous sucrose density gradient. Viral titration was performed in double on Vero cells. The titer was expressed as PFU/ml. Herpes Simplex type I virus (HSV-1), which was used as a non-specific control, was also grown on Vero cells infected with 0.01 PFU/cell for 1 h at 37 °C, and left to grow for three days before harvesting. After three cycles of freeze/thawing, the virus was sucrose gradient purified harvested, centrifuged as before, sonicated and titred. Different viral dilutions were prepared in PBS dilution buffer (PBS^-^, 0.2% Bovine Serum Albumin, 0.1% NaN_3_), starting from a concentration of 10^8^ PFU/ml down to 10^3^ PFU/ml. 200 μl were used for each determination. HSV-1 inactivation was performed by UV and psoralen. In brief, the virus (8 × 10^8^ PFU/ml) was mixed with a final psoralen concentration of 10 μg/ml. The mixture was incubated on a 6-well dish for 10 min at room temperature (1 ml/well) under UV lamps (366 nm) at 2 cm distance for 12 min (3 min each time with 1 min interval). Inactivation was tested by viral titration to verify the absence of infectious virus. All of the experiments were performed in a BSL-2 facility in the Laboratory of Virology at the State University of Milan (Italy), Dept. of Medical Biotechnologies and Translational Medicine.

Our baseline solution was created by mixing PBS + BSA (bovine serum albumin) 0.2% + NaN3 (sodium azide) 0.1%. Solutions with viruses were diluted in this base mixture, and the following viral concentrations were obtained: from 10^3^ to 10^8^ PFU (plaque forming units). In total, two viruses were used: alive vaccinia virus and herpes simplex virus 1 (UV-inactivated) as a control. Then solutions containing viruses were filtered through 0.22 μm filter paper inside the fume hood for sterility.

Further the response of the sensor over time was measured using the OBR, starting with a reference level of PBS and then gradually increasing the virus concentration from 10^3^ to 10^8^ PFU. The measurements were taken three times, with each measurement taken 20–30 s apart from each other. Each measurement was reported after stabilizing the sensor readout for about 10 min.

### Specificity

For specificity the BR sensors were functionalized by attaching specific antibodies for L1 and rabbit antibodies separately. As part of a control experiment, some sensors were exposed to the herpes simplex 1 virus. Out of five of the sensors marked with anti-L1 antibodies, two sensors were used to measure vaccinia, two sensors were used to detect herpes as a control, and one sensor was used to measure samples with vaccinia and herpes simultaneously at the same concentration. Two sensors were modified with rabbit antibodies and used to detect either vaccinia or herpes as a control. To account for differences in sensitivity among the sensors, the responses of each sensor to the virus measurements were adjusted by normalizing with the RI sensitivity for each sensor, resulting in an output that indicates the equivalent RI change.

### Detection limit computation

The limit of detection (LoD) was computed using the method reported by Chiavaioli et al.^35^. At first, the output of each sensor was compared to a reference fit using a log-quadratic regression, which corresponds to the wide-range behavior of BRs in PBS media^21^. The LoD was analytically computed as x_LoD_ = f^−1^(y_blank_ + 3σ_max_), where x_LoD_ is the concentration limit, y_blank_ is the sensor response for the reference level (evaluated at the lowest viral concentration), σ_max_ is the worst-case standard deviation computed over 3 consecutive measurements spaced by > 50 s), and y = f(x) is the log-quadratic input–output relation.

## Results and discussion

### Operation of ball resonators

In order to evaluate the possibility of detecting vaccinia virus in various operative contexts, 7 ball resonators have been fabricated. The operation of a ball resonator is shown in Figure S1, while a table showing all sensors and their functionalization process is shown in Table S1.

Seven sensors have been fabricated, having a ball diameter ranging from 518 to 569 μm, a having sensitivity ranging from 41.935 dB/RIU to 244.879 dB/RIU (average: 104.48 dB/RIU, standard deviation 70.50 dB/RIU), having ratings comparable to^32^. Five sensors were functionalized using anti-L1 antibodies; two sensors were used for vaccinia measurement, two sensors were used for herpes (control) detection, while one sensor was used for the measurement of vaccinia and herpes at same concentration. Two sensors were functionalized with rabbit antibodies, and used for the detection of either vaccinia or herpes control. In order to account for the different sensitivity value, the responses for each sensor throughout the virus measurement were normalized by the RI sensitivity for each sensor, obtaining an output that displays the equivalent RI change; the normalization was performed by diving the recorded intensity change in the optical spectrum by the estimated sensitivity for each sensor (as reported in Supplementary Table S1.

### Vaccinia virus detection using the anti-L1 antibodies

**Figure 3:**
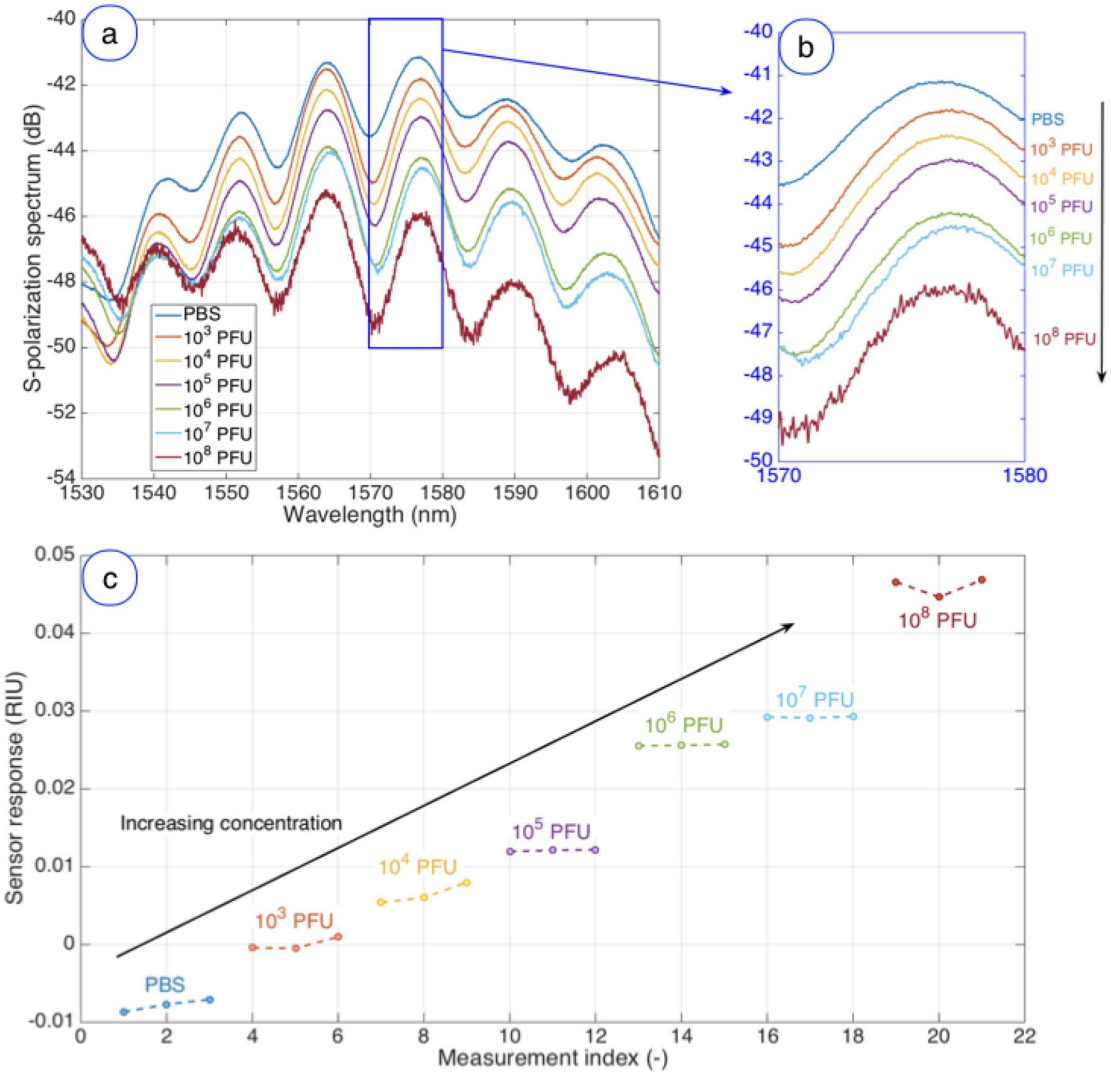
Detection of the vaccinia virus at various concentrations through a ball resonator functionalized with anti-L1 antibodies. (**a**) S-polarization spectrum, measured for each concentration of vaccinia from 10^3^ to 10^8^ PFU and for the reference value in PBS. (**b**) Inset on the highlighted blue region (1570–1580 nm) containing the most prominent spectral feature, i.e. the spectral peak around 1576.5 nm. (**c**) Timeline of the vaccinia virus detection, reporting the response of the sensor over time, for the reference level (PBS) and increasing the virus concentration from 10^3^ to 10^8^ PFU.

The process of detection of VV using a biosensor functionalized with anti-L1 antibody is displayed in Fig. 3. The spectral fingerprint of the sensor exhibits a set of peaks mainly located within 1550–1600 nm; by detecting the peak at around 1576.5 nm, we can observe a decreasing pattern, as the spectral intensity drops as the viral concentration increases. The reference value can be observed in PBS as − 41.17 dB. When the viral concentration at which he sensor is exposed is set to 10^3^ PFU (plaque forming units), the intensity of the spectral peak drops to − 41.81 dB. The, we can observe that the inner peak intensity is drops to − 42.42 dB, − 42.98 dB, − 44.21 dB, − 44.53 dB, and − 45.97 dB as the viral concentration is increased to 10^4^ PFU, 10^5^ PFU, 10^6^ PFU, 10^7^ PFU, and 10^8^ PFU respectively.

A sensorgram reporting the temporal evolution of the detection is displayed in Fig. 3c. In order to account for the sensitivity of the sensor, the output is normalized by the sensitivity (− 91.943 dB/RIU). For each concentration, the sensor output has been stabilized for 10 min, then 3 consecutive measurements spaced 15–35 s apart from each other have been taken and displayed in the chart. The reference value was set as the average value observed for the smallest vaccinia concentration (10^3^ PFU). The increase of concentration results in output change up to 46.86 × 10^−3^ RIU; we observe that the measurement has a good stability between each of the 3 measurements (standard deviation ranging from 0.19 to 2.79 mRIU).

### Vaccinia virus detection using rabbit serum antibodies

**Figure 4:**
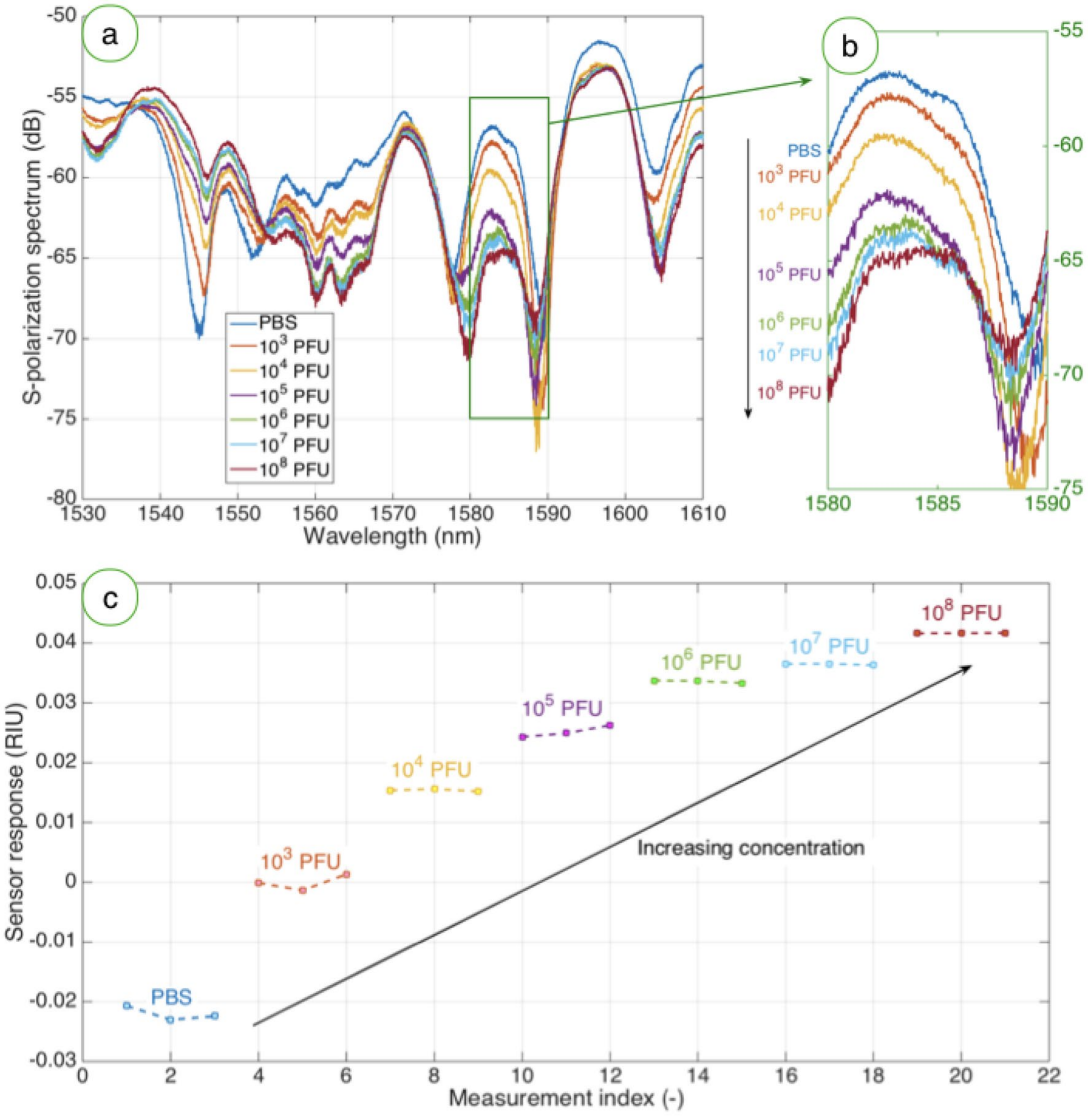
Detection of VV at different concentrations through a ball resonator functionalized with rabbit serum antibodies. (**a**) S-polarization spectrum, measured for each concentration of VV from 10^3^ to 10^8^ PFU and for for the reference value in PBS. (**b**) Inset on the highlighted green region (1580–1590 nm) containing the most prominent spectral feature, i.e. the spectral peak around 1583 nm. (**c**) Timeline of VV detection, reporting the response of the sensor over time, for the reference level (PBS) and by increasing the virus concentration from 10^3^ to 10^8^ PFU.

The detection of VV through a ball resonator biofunctionalized with the antibody synthesized from rabbits is shown in Fig. 4. The first chart shows the spectral fingerprint, which differs from the previous figure as each BR sensor has a quasi-random pattern that differs from the other sensors^32^. The spectral peak of around 1583 nm is used for the detection. Similarly to the previous sensor, we observe a decreasing pattern in the intensity level, as the intensity drops from − 56.93 dB observed in PBS to − 57.89 dB for 10^3^ PFU, and then progressively drops to − 64.75 dB for 10^8^ PFU. The larger intensity change observed for this sensor is due to the different sensitivity, which is equal to − 155.022 dB/RIU for this specific unit. The sensorgram displays a similar pattern, both in terms of a qualitative trend and in the range of normalized change (up to 41.63 mRIU for the highest concentration of VV). The detection of VV appears to yield similar results for both types of biofunctionalization, which increases the robustness of the system.

### Limit of detection and specificity analyses

**Figure 5:**
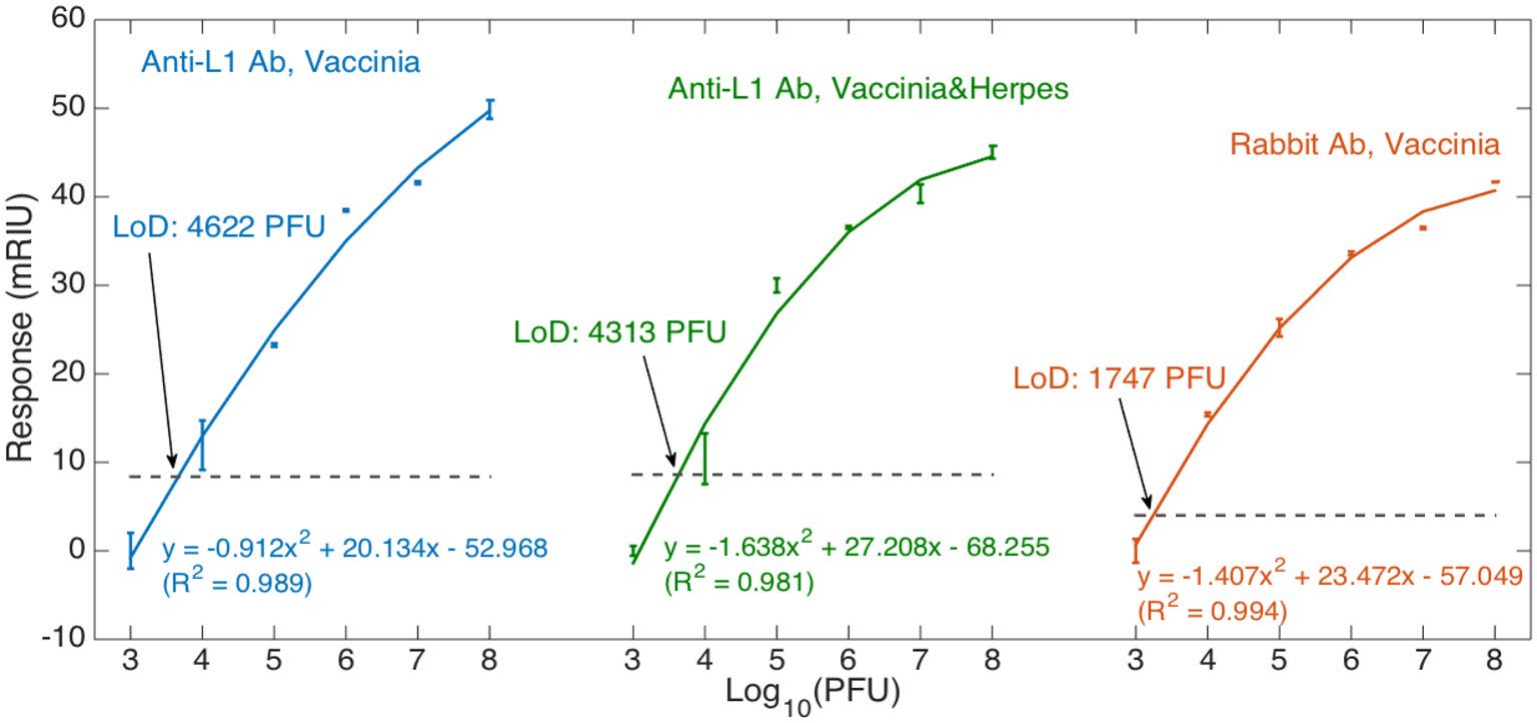
Response of different probes in the various measurement conditions, and limit of detection identification. The chart shows the sensor responses, obtained by normalizing the intensity change by the estimated sensitivity, for each concentration of vaccinia. Charts report the measured data (markers = average data; error bars =  ± standard deviation) and a log-quadratic fit obtained by polynomial regression (R^2^ > 0.98). Left/blue = probe functionalized with Anti-L1 antibodies measuring vaccinia; center/green = probe functionalized with Anti-L1 antibodies measuring vaccinia and herpes at the same concentration; right/red = probe functionalized with rabbit antibodies measuring vaccinia. Grey horizontal lines show, for each sensor, the limit of response y_LoD_ = y_blank_ + 3σ_max_ (standard deviation obtained through 3 consecutive measurements).

The Fig. 5 shows the response of three BRs, with different functionalization processes and working within different measurement scenarios: (1) Sensor functionalized with anti-L1 antibody, measuring vaccinia; (2) Sensor having the same anti-L1R biofunctionalization, measuring a mixture of VVand HSV-1 (control) at the same concentration; (3) Sensor with rabbit antibody-based functionalization. The three sensors show a similar output, both in terms of trend with respect to the viral concentration, and in the quantitative response (included within 0–50 mRIU for all sensors).

The response of the sensors has a logarithmic shape, following the trend of most of fiber-optic biosensors operating at a low concentration; the trend is similar for both BR sensors responding over a wide range of protein concentration^21^ as well as to most interferometric sensors detecting biomarkers^20^. The logarithmic response, similar to the one of potentiometric sensors based on Nernst relation applied to SARS-CoV-2 detection^36^, is a valuable asset for the design of a wide-range viral biosensor as it allows spanning a wide range of concentration, while maintaining a low detection limit.

The response of the sensors appears to have a steeper slope for low concentration, while slightly saturating towards the higher values; the behavior is very similar to a log-quadratic pattern, with coefficients of determination ranging within 0.981–0.994 for all the three cases. The individual coefficients obtained with quadratic regression are listed in Fig. 5, while the response for all sensors at low concentrations is larger than 20 mRIU for a two-order of magnitude change of viral concentration (10^3^ to 10^5^ PFU), as shown by the linear term in each input–output relation. The LoD was evaluated as 4622 PFU and 4313 PFU for the measurement with anti-L1R antibody functionalization, respectively in VV and VV/HSV-1 mixture. The LoD was estimated as 1747 PFU for the BR functionalized with rabbit serum antibodies. The similar response obtained for the three sensing units displays the robustness of the sensing system: since we observe a similar pattern for the VV and VV/HSV-1 cases, we can confirm that the sensor is specific to the target pathogen, and the selection of the antibody does not modify the optical behavior of the sensing unit. In proximity of the LoD, the sensitivity for a tenfold concentration change (10^4^–10^5^ PFU) is 11.93 mRIU for the anti-L1R sensor in VV, 12.46 mRIU for the anti-L1R sensor measuring VV and HSV-1, and 10.81 mRIU for the rabbit serum antibody sensor.

The detection limit that is obtained with the proposed fiber-optic sensor is well comparable with previous methods that have been proposed for VV detection, which include electroreduction-based electrochemical sensor for voltametric analysis proposed by Park et al.^37^ and achieving LoD of 4000 PFU/mL, as well as optical methods based on a labeled sandwich immunoassay for fluorescence detection proposed by Donaldson et al.^38^ (LoD: 2.5 × 10^5^ PFU/mL), and nanoplasmonic biosensors such as the one reported in 2010 from Yanik et al.^39^ achieving LoD ≤ 10^5^ PFU/mL.

**Figure 6:**
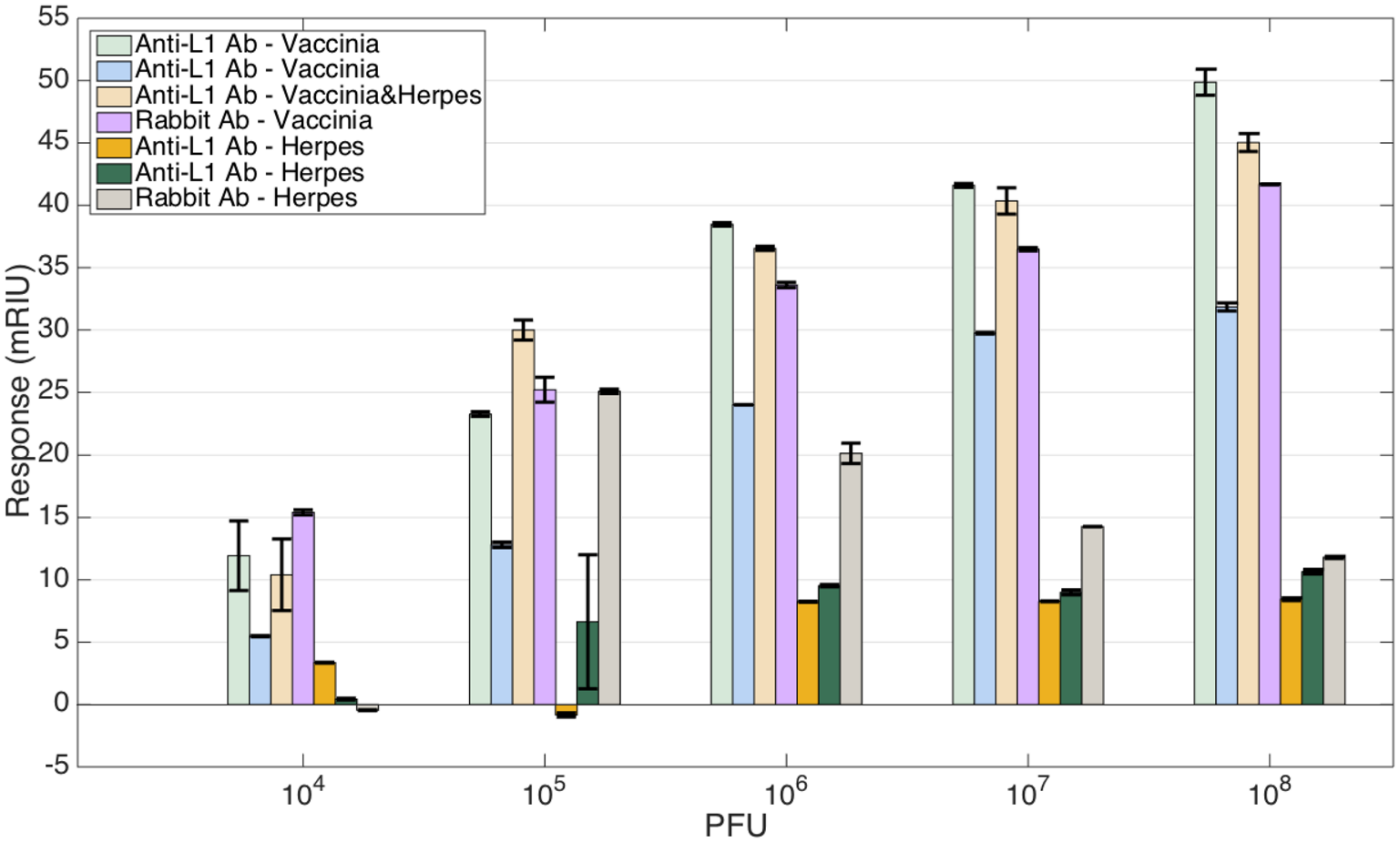
Comparison of the detection of vaccinia and specificity to herpes virus for all probes. For each vaccinia, herpes, or vaccinia/herpes mixture concentration ranging from 10^4^ to 10^8^ PFU the chart shows the average response from reference level (error bars =  ± standard deviation). The data report four sensors used for measurements of vaccinia (respectively, two sensors with anti-L1 antibodies measuring vaccinia, one sensor with anti-L1 antibodies measuring vaccinia/herpes mixture, and one sensor with rabbit antibodies measuring vaccinia), and three sensors for control reporting the response to herpes virus (respectively, two sensors with anti-L1 antibodies measuring herpes, and one sensor with rabbit antibodies measuring herpes).

A specificity analysis is included in Fig. 6, where we report the response obtained for each BR sensor, measuring either VV, or VV/HSV-1 combination, or HSV-1 alone, as a control. We display the normalized responses obtained for each sensor, in order to compensate the difference in RI sensitivity for each device. All the sensors designed for VV detection exhibit a log-quadratic pattern. A different sensor functionalized with anti-L1R antibodies exhibits a similar response, but with a lower slope, possibly due to the different size of the device and sensitivity, while still showing a response > 30 mRIU at 10^8^ PFU concentration. The data reported for HSV-1 exhibits a different trend: for the anti-L1R antibodies, the response is lower than 10 mRIU at concentrations up to 10^7^ PFU. At the highest concentration, the responses obtained for anti-L1R antibodies are 31.8–49.9 mRIU for VV, 45.0 mRIU for VV/HSV-1, and 8.4–10.6 mRIU for the HSV-1 control.

A different pattern was observed for the rabbit serum antibodies: hereby the response related to HSV-1 control rises to 25.1 mRIU at 10^5^ PFU, and then reduces to 11.8 mRIU at 10^8^ PFU, showing a pattern without a clear rising trend. Overall, this approach appears to have a quite similar specificity with respect to anti-L1 antibodies at the highest concentration. The response to HSV-1 measured for rabbit serum antibodies at 10^8^ PFU is 28.3% lower than the response to VV, compared to 21.2% for anti-L1 antibodies (accounting for the largest responses), but the specificity at intermediate concentrations is lower. Although no binding should be obtained with HSV, a limited non-specific binding could be expected. Further experiments should be performed to explain the discrepancy of the inverted trend resulting by increasing viral PFU.

## Conclusions

In this work, we reported the design of label-free biofunctionalized ball resonator fiber-optic sensors for the detection of a pandemic poxvirus through a wide range of viral concentrations. We compared a mouse monoclonal anti-L1R antibody that binds to the L1R protein expressed by poxviruses andpolyclonal antibodies elicited in rabbits that target the whole virus particle. Both showed similar performances, with responses up to 31.8–49.9 mRIU for the corresponding changes of concentration of VV from 10^3^ to 10^8^ PFU. All sensors exhibited a log-quadratic response, with higher sensitivity towards the lowest concentration. The LoD for all sensors was 1747–4313 PFU, while the sensitivity around the LoD was 10.81–12.46 mRIU for a 10 × concentration change. The anti-L1R antibody provides a more robust sensing method, as confirmed both by the specificity analyses as the response in HSV-1 control is up to 21.2% lower than the response to VV, and by the very similar trend shown when comparing the response in VV and in VV/HSV-1 mixture.

The proposed method reports for the first time the detection of an orthopoxvirus in liquid through a simple label-free fiber optic biosensor, that exhibits rapid and stable detection across a wide range of virus concentration, low detection limit, and the possibility to operate remotely. The results obtained for viral detection have a very strong analogy with the results reported for the detection of protein-based cancer biomarkers^21^. In addition, BR sensors are suitable to be multiplexed into larger sensing networks, exploiting the spatial dimension provided by the OBR interrogator^40^.

The possibility to detect viral pathogens in a wide concentration range and with a low-cost disposable sensor is a first step towards the “pandemic watchdog” system proposed for wastewater monitoring during the COVID-19 pandemic^8^ and that is rapidly taking interest for early-stage detection of viral outbreaks^41^. Further work will aim at the measurement of orthopoxviruses in different analytes, at the discrimination between viral particles and proteins shed into the analyte, as well as on the demonstration of the deployment of a large-scale sensing network.

### Supplementary Information

Supplementary Information.

## Data Availability

The datasets for the present investigation (raw data and processed files) are available from the corresponding author upon reasonable request.
