# Supplementary material for: Rapid detection of vaccinia virus using biofunctionalized fiber-optic ball-tip biosensors
Source: Sci Rep. 2023 Oct 14;13:17470. doi: 10.1038/s41598-023-44926-6 (PMC10576743; doi:10.1038/s41598-023-44926-6)
Supplement: Supplementary file 1 — Supplementary Information. [file 41598_2023_44926_MOESM1_ESM.docx]

**Rapid detection of vaccinia virus using biofunctionalized fiber-optic ball-tip biosensors**

Aida Rakhimbekova^1^, Baizak Kudaibergenov^1^, Kuanysh Seitkamal^2^, Aurora Bellone^3^, Ayazhan Dauletova^1^, Marzhan Sypabekova^4^, Massimo Olivero^3^, Guido Perrone^3^, Antonia Radaelli^5.6^, Carlo Zanotto^5^, Carlo De Giuli Morghen^6^, Luca Vangelista^2,7^, Daniele Tosi^1,8,*^

^1^ Department of Electrical and Computer Engineering, School of Engineering and Digital Sciences, Nazarbayev University, 010000 Astana, Kazakhstan

^2^ Department of Biomedical Sciences, School of Medicine, Nazarbayev University, 010000 Astana, Kazakhstan

^3^ Department of Electronics and Telecommunications, Politecnico di Torino, Turin, Italy

^4^ Department of Electrical & Computer Engineering, Baylor University, Waco, TX, USA

^5^ Department of Medical Biotechnologies and Translational Medicine, Laboratory of Molecular Virology and Recombinant Vaccine Development, University of Milan, via Vanvitelli 32, Milan, Italy

^6^ Catholic University “Our Lady of Good Counsel”, Rr. Dritan Hoxha, Tirana, Albania

^7^ Department of Molecular Medicine, University of Pavia, 27100 Pavia, Italy

^8^ Laboratory of Biosensors and Bioinstruments, National Laboratory Astana, 010000 Astana, Kazakhstan

**Supplementary materials**


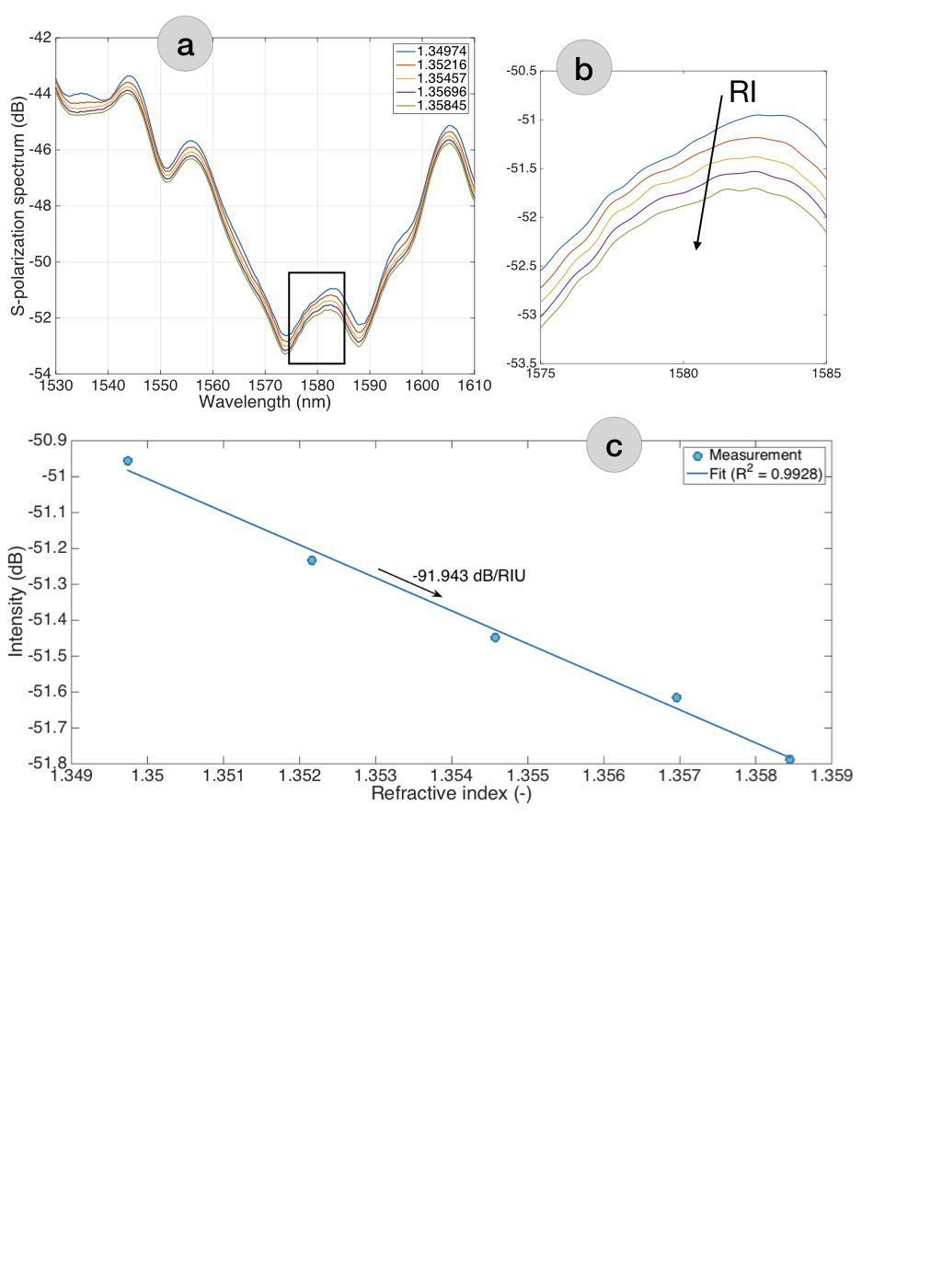


**Figure S1**. Refractive index sensitivity of a ball resonator. (a) S-polarization spectrum for a ball resonator of size 518-513 μm, reporting the reflection spectrum for RI values ranging from 1.34974 to 1.35845. (b) Inset on the analyzed spectral feature, with reflection peak reducing the intensity as the RI increases. (c) Evaluation of the sensitivity to RI, as the estimated (linear regression) intensity variation for each RI value. The RI sensitivity for this sensor is -91.943 dB/RIU (R^2^ = 0.9928).

| **Sensor number** | **x-y diameter (μm)** | **RI sensitivity (dB/RIU)** | **Bioreceptor** | **Detection** |
| --- | --- | --- | --- | --- |
| 1 | 518-513 | -91.943 | Anti-L1 Ab | Vaccinia |
| 2 | 531-521 | -71.905 | Anti-L1 Ab | Vaccinia |
| 3 | 569-563 | -90.573 | Anti-L1 Ab | Vaccinia+Herpes |
| 4 | 564-556 | -175.534 | Anti-L1 Ab | Herpes |
| 5 | 565-560 | -41.935 | Anti-L1 Ab | Herpes |
| 6 | 533-523 | -155.022 | Rabbit Ab | Vaccinia |
| 7 | 545-535 | -244.879 | Rabbit Ab | Herpes |

**Table S1.** List of the sensors used for the detection of viruses. The table reports: the diameter measured by the CO_2_ laser splicer using the profilometry routine (±1 μm) on the xy plane perpendicular to the fiber orientation; the RI sensitivity evaluated on the perpendicular (S) polarization for all sensors by tracking the most significant spectral feature; the antibody immobilized as bioreceptor; the target pathogen for detection. Sensitivity data have been used for normalizing the sensor outputs.


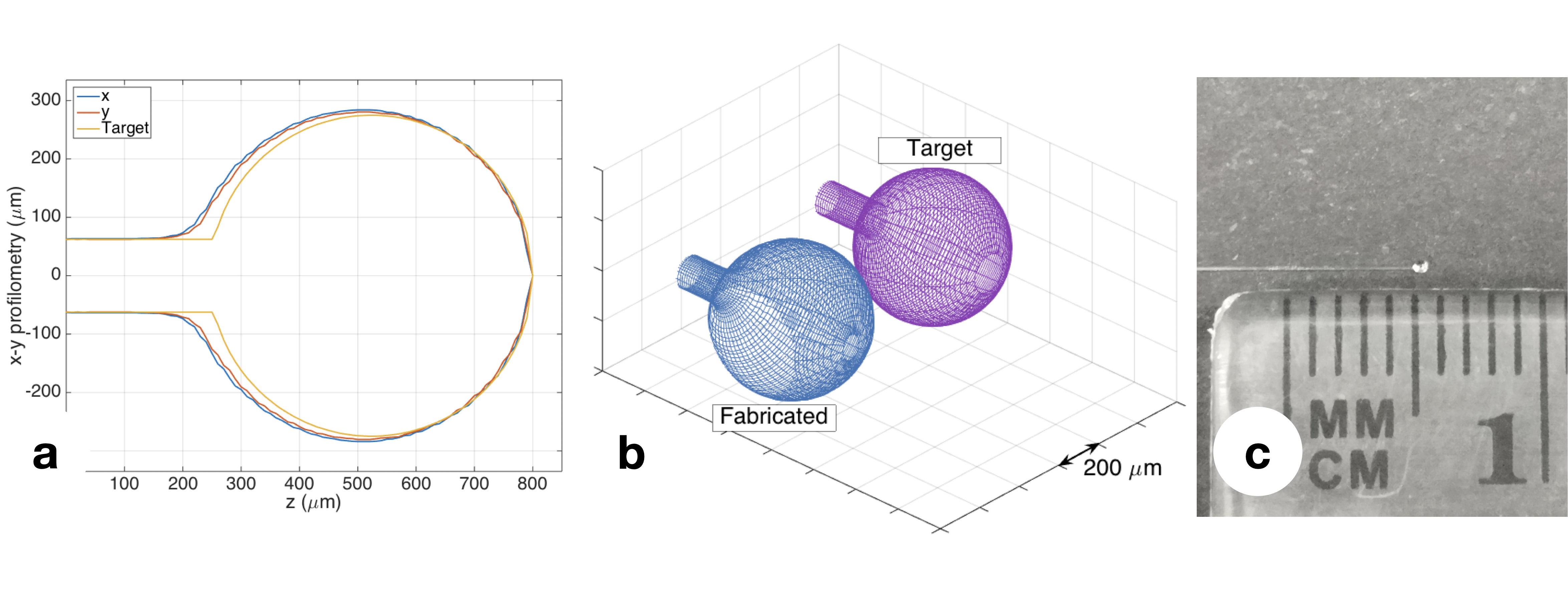


**Figure S2.** Ball resonators fabrication: (a) BRs profilometry; (b) BRs 3D modeling view; (c) BR view with 500μm diameter.

**Enzyme‑linked immunosorbent assay (ELISA)**

The rabbit plasma samples from T0 to T5 were assayed for antibodies against heat inactivated vaccinia virus using enzyme-linked immunosorbent assays (ELISAs). VV was resuspended in 0.05 M carbonate-bicarbonate buffer, pH 9.6 (15 mM Na_2_CO_3_, 35 mM NaHCO_3_, 0.2% NaN_3_) and plated in 96-well microtiter plates (MaxiSorp; Nunc, Thermoscientifc, Roskilde, Denmark), 50 ul/well. The antigen was incubated overnight at 4  °C at 10^5^ PFU/well. After washing 6 times with wash buffer (0,05% Tween 20 in PBS^-^), the plate was saturated with 3% skim milk in PBS^-^ (200 µL/well) for 1 h at 37 °C and washed again. Sera dilutions were prepared in 0,3% skim milk in wash buffer, starting from 1:1,000 dilution up to 1:300,000 depending on serum concentration and incubated for 1 h at 37 °C. After washing as before, the secondary goat anti-rabbit mouse horseradish-peroxidase-conjugated (HRP-conjugated) antibody was used (dilution, 1:2,000; DakoCytomation, Santa Clara, CA) for 1.5 h at 37 °C. The reactions were revealed after incubation for 30 min with tetramethylbenzidine substrate (TMB, Sigma–Aldrich) in the dark. The reaction was stopped by addition of 2 M H_2_SO_4_ (25 ul/well). The pre-immune mouse sera (T0) were used as the negative controls. The absorbance of each well was read at 450 nm using a microplate reader (550; Bio-Rad, Hercules, CA, USA). ELISAs were performed in duplicate.
